# Bruxism as a Biopsychosocial Disorder: An Interdisciplinary Cross-Sectional Study

**DOI:** 10.3390/jcm14196803

**Published:** 2025-09-26

**Authors:** Karolina Walczyńska-Dragon, Dominika Grzybowska-Ganszczyk, Paweł Hadzik, Jakub Fiegler-Rudol, Izabela Dubiel-Holecko, Aleksandra Nitecka-Buchta, Stefan Baron

**Affiliations:** 1 Department of Temporomandibular Disorders, Medical University of Silesia in Katowice, Traugutta Sq. 2, 41-800 Zabrze, Poland; aleksandra.nitecka@sum.edu.pl (A.N.-B.); sbaron@sum.edu.pl (S.B.); 2 Department of Clinical Physiotherapy, Academy of Physical Education in Katowice, 72B Mikołowska Street, 40-065 Katowice, Poland; d.grzybowska-ganszczyk@awf.katowice.pl; 3 Department of Physiotherapy, Opole University of Technology, Prószkowska Street 76, 45-758 Opole, Poland; pawel.hadzik@student.po.edu.pl; 4 Student Scientific Society at the Department of Temporomandibular Disorders, Medical University of Silesia in Katowice, 41-800 Zabrze, Poland; s88998@365.sum.edu.pl; 5 Institute of Psychology, Department of Social Sciences, University of Silesia in Katowice, Grażyńskiego 53, 40-126 Katowice, Poland; izabela.dubiel@us.edu.pl

**Keywords:** anxiety symptoms, bruxism, coping strategies, pain, psychoemotional state, temporomandibular disorders

## Abstract

**Background**: Bruxism is a multifactorial biopsychosocial condition involving repetitive jaw muscle activity, influenced by psychological stress, anxiety, and maladaptive coping strategies. **Methods**: In this cross-sectional observational study, 111 health sciences students (mean age 22.29 ± 3.10 years; 66.6% women) underwent standardized DC/TMD clinical evaluation and completed the GAD-7, COPE Inventory, and a 105-item Stroop Test. **Results**: Bruxism prevalence was 63.96% (71/111). Students with bruxism had higher GAD-7 scores (10.63 ± 5.78 vs. 5.80 ± 3.66; mean difference 4.83, 95% CI 2.88–6.78; *p* < 0.001, g = 0.94), with clinically relevant anxiety nearly three times more frequent (55.4% vs. 19.6%; RR = 2.83, 95% CI 1.68–4.76). Stroop interference times were slower in the bruxism group (42.19 ± 16.87 s vs. 34.57 ± 16.25 s; mean difference 7.63 s, 95% CI 0.90–14.35; *p* = 0.027, g = 0.46), though accuracy was similar. COPE results showed a shift toward emotion-focused and avoidance strategies with increasing muscle pain. **Conclusions**: Bruxism is strongly linked to elevated anxiety, maladaptive coping strategies, and reduced inhibitory control speed. Integrating psychological screening tools such as GAD-7 and COPE into dental and medical practice could enable early identification and referral, supporting comprehensive management that combines dental treatment with psychological interventions to prevent chronic pain and long-term complications.

## 1. Introduction

Bruxism represents a complex, multifaceted phenomenon that has emerged as a significant clinical challenge requiring interdisciplinary attention from healthcare professionals across multiple specialties [1,2]. It is defined as repetitive jaw-muscle activity that may occur during sleep or wakefulness, characterized by distinct circadian patterns and specific muscle activation profiles [3,4]. Contemporary understanding recognizes psychosocial influences as central to both the taxonomy and clinical expression of bruxism, reflecting its complex and multifactorial nature [5]. Within university populations, awareness of bruxism’s causes, consequences, and therapeutic approaches varies widely, indicating differences in risk perception and patterns of help-seeking behavior [6,7,8,9,10]. Prevalence estimates for awake bruxism in adults are substantial, underscoring its significance as a public health concern [11,12,13,14,15,16,17,18]. The pathophysiology of bruxism involves central neural circuits, with evidence emerging from neuroimaging and electrophysiological studies, alongside epidemiologic associations with psychological load [6,7]. Clinical and hematologic predictors observed in temporomandibular disorder cohorts further support the concept of a stress-sensitive phenotype [8]. Dysregulation of the body’s stress systems appears to play a pivotal role, particularly through hyperactivation of the hypothalamic–pituitary–adrenal axis and autonomic nervous system. This is supported by findings of altered salivary cortisol levels and maladaptive coping patterns in affected individuals, as well as by reviews emphasizing the importance of stress-focused interventions in bruxism management [9,10,11,12]. Limbic structures, especially the amygdala, are likely to contribute to heightened threat perception and increased motor readiness, which may manifest as excessive activation of the masticatory muscles [16]. Cognitive mechanisms are also relevant to understanding bruxism. Executive functions, particularly inhibitory control, are crucial for regulating automatic or maladaptive motor behaviors. Individual differences in Stroop task performance reflect the integrity of these processes, with impaired inhibitory control linked to difficulties suppressing parafunctional activities such as bruxism [13]. Environmental stressors can further disrupt cognitive performance, highlighting the interaction between psychological stress and neurocognitive regulation of motor output [19,20,21]. Collectively, current research supports a multifactorial, biopsychosocial model of bruxism, with interdependent biological, psychological, and social factors shaping its onset, severity, and clinical course [22].

Despite this growing body of knowledge, important gaps remain. Many studies focus on either anxiety, coping strategies, or executive control in isolation and rely primarily on self-reported bruxism rather than standardized clinical evaluation. Few have integrated clinical dental examinations with validated measures of anxiety severity, detailed coping profiles, and performance-based assessments of executive function within a single, well-characterized cohort. This limitation is particularly notable among health-sciences students, who experience distinctive academic and clinical stressors that may influence both symptom development and cognitive performance. Addressing this gap, the present cross-sectional study combines standardized orofacial assessment with the Generalized Anxiety Disorder Scale (GAD-7), the COPE Inventory, and the Stroop task to examine the complex interplay between anxiety, coping, and inhibitory control in this population [23,24,25,26,27,28]. We hypothesized that students with clinically confirmed bruxism would exhibit higher anxiety severity, greater reliance on emotion-focused or avoidance coping strategies, and longer Stroop interference times while maintaining accuracy compared to peers without bruxism. This study is novel in integrating clinical bruxism assessment with validated anxiety, coping, and cognitive inhibition measures, revealing that bruxism is linked not only to heightened anxiety and maladaptive coping but also to subtle executive control deficits, offering a more comprehensive understanding of its biopsychosocial mechanisms.

## 2. Bruxism

According to the 2018 international consensus, bruxism is a repetitive jaw-muscle activity manifested as clenching, grinding, tensing, or thrusting of the mandible [3]. It is classified into sleep bruxism (SB) and awake bruxism (AB). The 2025 consensus further defined their circadian and muscular features: SB occurs during sleep with rhythmic (phasic) or non-rhythmic (tonic) activity, while AB occurs during wakefulness with repetitive or sustained tooth contact, bracing, or thrusting [3,4]. Bruxism is now considered as a behavioral sign rather than a disease, acting as either a risk or protective factor depending on context [4]. Tooth-contact activities such as clenching or grinding increase risks of dental wear, fractures, sensitivity, and compromised restorations [29]. Isometric activities, even without tooth contact, contribute to temporomandibular disorders, myofascial pain, and headache [12]. Conversely, bruxism may play protective or adaptive roles, as suggested by associations with obstructive sleep apnea (OSA) and gastroesophageal reflux disease (GERD) [30]. Epidemiologically, AB affects ~16–32% of the population, while SB occurs in ~8–16% of adults [31]. These figures vary widely due to differing diagnostic methods. Historically viewed as a neuromuscular disorder driven by genetic, occlusal, and neurochemical factors, bruxism is now linked to central nervous system dysregulation, especially of dopaminergic and serotonergic pathways [4]. Stimulants such as caffeine, nicotine, and certain drugs can exacerbate symptoms via CNS activation [32]. Psychoemotional factors are central to bruxism etiology. Stress, anxiety, and emotional disturbances are strongly correlated with both SB and AB [22]. Stress-induced sympathetic activation raises muscle tone, producing repetitive jaw movements [33]. Polysomnography shows that SB episodes often follow microarousals with increased heart rate variability, highlighting the role of stress and sleep fragmentation [34]. Personality traits such as perfectionism, neuroticism, and somatization, along with maladaptive coping styles, reinforce chronicity [35]. A bidirectional relationship exists: stress triggers bruxism, while bruxism-related pain and dental damage heighten distress, perpetuating a cycle. Therapies targeting psychoemotional factors, such as psychotherapy, relaxation training, and biofeedback, have been shown to alleviate symptoms and reduce pain [36]. These findings highlight the importance of a multidisciplinary approach that combines dental, medical, and psychological expertise.

## 3. Methodology

### 3.1. Study Design

The objective of this study was to investigate the relationships between clinically assessed bruxism, anxiety severity, coping strategies, and executive inhibitory control in health sciences students, providing a comprehensive understanding of the biopsychosocial mechanisms underlying bruxism symptoms.

This research was designed as a cross-sectional observational study aimed at examining the relationships between clinically assessed bruxism, anxiety severity, coping strategies, and executive inhibitory control in health sciences students. Participants were recruited from the Department of Temporomandibular Disorders, Zabrze, Medical University of Silesia; the Department of Physiotherapy, Opole, University of Technology; and the Department of Theoretical and Practical Foundations of Physiotherapy, Academy of Physical Education, Katowice. Ethical approval was obtained from the Bioethical Committee of the Medical University of Silesia (decision no. BNW/NWN/0052/KB/142/25, issued on 28 April 2025). All participants received both verbal and written information about the study and provided informed consent prior to participation. Patients enrolled in the study met the selection criteria outlined in Table 1.

The gold standard for the diagnosis of sleep bruxism involves polysomnography (PSG) with simultaneous audio-video recording, while electromyographic (EMG) monitoring provides objective data on jaw-muscle activity during wakefulness [3,4,31]. However, these methods are resource-intensive, time-consuming, and often impractical in large-scale population studies or in non-clinical samples such as university students. Therefore, in accordance with the 2018 international consensus on bruxism assessment, which recognizes different levels of diagnostic certainty, we adopted a clinical diagnosis of probable bruxism [3,28,37]. This approach, while less definitive than PSG or EMG, is widely applied in epidemiological and interdisciplinary research and provides a feasible, standardized method for identifying individuals at elevated risk of bruxism-related complications.

We followed the 2018 and 2025 international consensus statements, which distinguish three levels of diagnostic certainty: possible, probable, and definite bruxism [3,4]. In our non-laboratory, population-based setting we targeted probable bruxism by combining two complementary approaches: (1) a standardized clinical DC/TMD-based orofacial examination by a TMD-experienced dentist, and (2) a DC/TMD-derived self-report of bruxism-related behaviors and symptoms. Definite bruxism would require PSG or EMG, which are impractical for large student samples and could introduce selection bias. To limit misclassification, the clinical examiner was trained before the study, assessments followed the Polish DC/TMD instruments.

Participants were classified as having probable bruxism if they met the predefined clinical criteria and reported consistent symptoms. Those who did not meet these combined criteria were placed in the non-bruxism group, which served as the control group. Of the 111 enrolled participants, 71 (63.96%) were classified as having bruxism and 40 (36.04%) as non-bruxism. The sample size calculation was conducted post hoc using the effect sizes observed in the study. While this confirmed adequate statistical power for primary outcomes, it represents a limitation because no a priori sample size determination was performed. This dual-method approach (structured self-report plus standardized clinical exam) aligns with recommendations to use at least two convergent sources when PSG or EMG are not feasible.

### 3.2. Sample Size Calculation

A post hoc sample size calculation was conducted to assess the adequacy of the study cohort. Based on the effect sizes observed in the regression models, the current sample provided statistical power (>80%) to detect differences of this magnitude at an alpha level of 0.05. This suggests that the number of participants included was adequate for the primary analyses performed. A post hoc assessment indicated that with n1 71 and n2 40, two-sided alpha 0.05, the study had power > 0.99 to detect the observed GAD-7 effect (d ≈ 0.94) and approximately 0.80 to detect the observed Stroop interference effect (d ≈ 0.46). These values describe sensitivity for our primary contrasts and do not alter the inferential results.

### 3.3. Bruxism Diagnosis and Functional Orofacial Assessment

A dentist experienced in orofacial pain and TMD conducted a detailed clinical orofacial evaluation for all participants. The assessment included examination of masticatory muscle pain, temporomandibular joint (TMJ) clicking or dysfunction, dental wear patterns indicative of sleep bruxism (SB), abnormal mandibular movement trajectories, and the limited range of mandibular movements. Probable bruxism diagnosis was based on functional clinical examination and patients self-report, according to DC/TMD classification [37]. Following Professor Baron’s classification [28], participants were classified as bruxers if at least three of the following were present: recurrent or morning headaches, pain in masseter or TMJ during function, TMJ clicking or restricted motion, tooth wear suggestive of sleep bruxism, and tenderness on palpation of masticatory muscles. Assessments followed the Polish version of the DC/TMD criteria [37]. The evaluation was based on palpation tenderness and Baron’s indicators, using a 0–1 scale, without assessing the severity of bruxism.

### 3.4. Psychoemotional and Cognitive Evaluation

Participants completed the following validated assessment tools:

- COPE Inventory: A 60-item self-report tool measuring 15 coping strategies across adaptive, maladaptive, and context-dependent domains, rated on a 4-point Likert scale [38]. The Polish adaptation shows good reliability (α = 0.72–0.88; total α = 0.86) [39].
- Generalized Anxiety Disorder Scale (GAD-7): A 7-item self-report tool screening generalized anxiety on a 0–3 scale, with total scores ranging 0–21 [40]. The Polish version demonstrates high reliability and validity (α = 0.92) [41].
- DC/TMD Bruxism Symptoms Questionnaire: Assessing subjective severity and frequency of bruxism-related symptoms. It is a self-report instrument derived from the Diagnostic Criteria for Temporomandibular Disorders (DC/TMD), designed to evaluate patients’ perception of bruxism behaviors and their clinical relevance [37].
- Stroop Test (105-item version): The Polish version of the Stroop Color and Word Test (SCWT) was used to assess executive function, cognitive inhibition, and selective attention [42,43]. The task included three conditions with 105 randomized stimuli each: (1) word reading (color names in black), (2) color naming (colored squares), and (3) color-word naming (incongruent words and ink colors). In the incongruent condition, participants were required to suppress the automatic reading response in favor of naming the ink color. Reaction times and both corrected and uncorrected errors were recorded. Cognitive interference was calculated by comparing incongruent (CW) performance with word (W) and color (C) conditions. The test is widely validated and frequently applied in both research and clinical assessment of inhibitory control [44,45].

### 3.5. Control Group Definition

Participants were allocated to two analytic groups based on the clinical bruxism assessment described below. The bruxism group comprised participants meeting the a priori criteria for probable bruxism. The control group comprised participants who did not meet those criteria on the same standardized examination and self-report screen. Controls were drawn from the same source population, recruited during the same period, and underwent identical eligibility screening, DC/TMD exam, and questionnaires. No matching was used; group differences were addressed analytically with effect sizes, confidence intervals, and multivariable models.

### 3.6. Statistical Analysis

Statistical analysis involved descriptive statistics and comparisons between groups to explore relationships between bruxism severity, psychoemotional factors, coping strategies, and cognitive functioning. SPSS software (version 27) was used, with significance set at *p* < 0.05. Continuous variables were summarized as mean ± SD or median (IQR), depending on distribution, and categorical variables as n (%). Normality was assessed using the Shapiro–Wilk test and visual inspection of Q-Q plots. For two-group comparisons, independent-samples t-tests were applied when parametric assumptions were met, otherwise Mann–Whitney U tests were used. For comparisons across multiple groups, one-way ANOVA or Kruskal–Wallis tests were performed. Categorical variables were compared using chi-square or Fisher’s exact tests. Effect sizes were calculated for all comparisons: Hedges g with 95% CI for mean differences, Cliff’s delta for nonparametric two-group contrasts, epsilon-squared for Kruskal–Wallis tests, and Cramér’s V for chi-square tests. For categorical outcomes, risk differences and risk ratios with 95% CIs were also reported. Associations between ordinal or non-normally distributed variables were assessed with Spearman’s rho. All analyses were prespecified before unblinding group labels.

## 4. Results

### 4.1. Participants, Bruxism Diagnosis and Classification

The sample comprised 111 adults (*n* = 111), aged 18–37. The study group includes women (67.3%) and men (32.7%). Most participants are between 18 and 26 years old (91.8%), followed by smaller groups aged 27 to 30 (6.4%) and 31 to 40 (1.8%). When looking at specific ages, the most common are 19 years old (17.8%), 23 years old (16.9%), 24 years old (15.7%), 22 years old (13.5%), and 20 years old (12.2%). Other ages include 21 (5.1%), 25 (4.1%), 26 (3.2%), 27 (5.5%), and 30 (2.4%), with tiny proportions at other ages. Regarding their field of study, 50.9% are enrolled in dentistry, 40% in biological renewal, and 9.1% in physiotherapy. In the dataset (*n* = 111), bruxism occurred in 63.96% of cases (*n* = 71), while no bruxism occurred in 36.04% of cases (*n* = 40). Table 2 and Table 3 present details about the study group.

### 4.2. General

#### 4.2.1. Anxiety Severity in Bruxism

**Figure 1:**
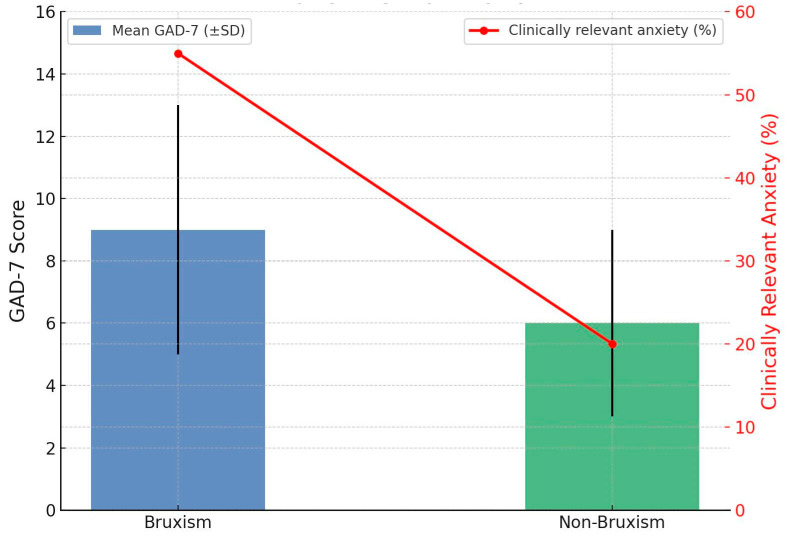
GAD-7 Scores and clinically Relevant anxiety in bruxism vs. non-bruxism.

Participants with bruxism demonstrated significantly higher GAD-7 scores than non-bruxism participants (10.63 ± 5.78 vs. 5.80 ± 3.66; *p* < 0.001, Hedges’ g = 0.94). Clinically relevant anxiety (GAD-7 ≥ 10) was nearly three times more common in the bruxism group (55.4%) than in controls (19.6%). Figure 1 compares anxiety levels between participants with bruxism and those without. The blue bars represent the mean GAD-7 scores with error bars showing standard deviation. Participants with bruxism had a significantly higher mean score (10.63 ± 5.78) than the non-bruxism group (5.80 ± 3.66). The red line shows the percentage of participants with clinically relevant anxiety (GAD-7 ≥ 10). This was nearly three times higher in the bruxism group (55.4%) compared to controls (19.6%). Overall, the figure demonstrates a clear association between bruxism and elevated anxiety symptoms (Figure 1).

#### 4.2.2. Executive Control (Stroop Interference)

**Figure 2:**
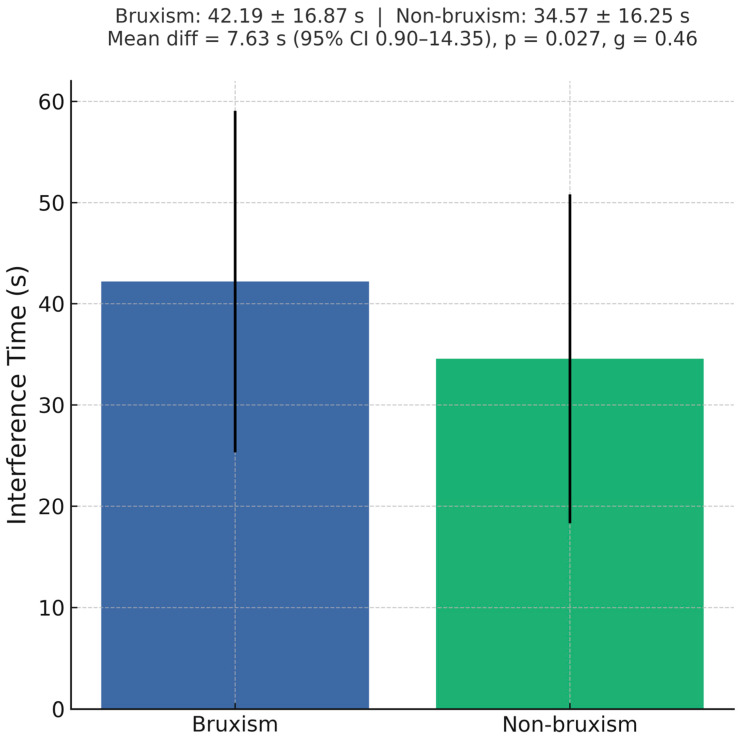
Stroop task interference time in bruxism vs. non-bruxism.

Bruxism was associated with reduced performance on the Stroop task. Interference time was significantly greater in the bruxism group (42.19 ± 16.87 s) compared with controls (34.57 ± 16.25 s), with a mean difference of 7.63 s (95% CI 0.90–14.35; *p* = 0.027, g = 0.46), indicating slower cognitive inhibition in individuals with bruxism (Figure 2). Relative and ratio interference indices followed the same direction but did not reach statistical significance. No significant correlation was observed between anxiety severity (GAD-7 total score) and interference time (r = 0.16, *p* = 0.11).

#### 4.2.3. Headache and Anxiety

Participants reporting temporal headaches in the preceding 30 days had significantly higher GAD-7 scores than those without (11.38 ± 5.06 vs. 6.86 ± 3.96; *p* = 0.010, g = 0.93). This association persisted after adjusting for bruxism status.

### 4.3. COPE Inventory

**Figure 3:**
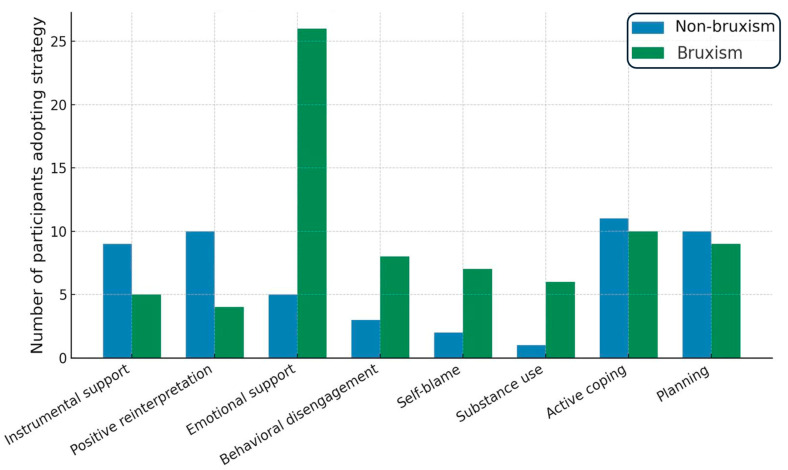
Coping strategies in bruxism vs. non-bruxism.

Coping strategy patterns also varied according to masticatory muscle pain severity. Kruskal–Wallis analyses showed significant effects for active coping (H(3) = 8.38, *p* = 0.039), planning (H(3) = 8.83, *p* = 0.032), focusing on and venting emotions (H(3) = 13.61, *p* = 0.0035), and behavioral disengagement (H(3) = 10.25, *p* = 0.0166). Post hoc tests indicated that participants with no pain reported significantly higher active coping scores than those in moderate pain (group 2; *p* = 0.038) and higher planning scores than group 2 (*p* = 0.050). Conversely, focusing on and venting emotions was more frequent in participants with moderate (group 2; *p* = 0.029) and severe pain (group 3; *p* = 0.0068) than those without pain. Behavioral disengagement was most pronounced in the severe pain group, which scored higher than the moderate pain group (*p* = 0.049). Overall, these findings indicate a shift from problem-focused strategies toward emotion-focused and avoidance-oriented coping as masticatory muscle pain severity increases. Analysis of COPE Inventory responses revealed differences in the types of coping strategies most frequently chosen by participants. In the no-bruxism group, the most common strategies were instrumental support and positive reinterpretation and growth, each reported by 9 participants. In contrast, the bruxism group most frequently endorsed use of emotional support, selected by 26 participants. A chi-square test of independence (χ^2^ = 19.76, df = 12, *p* = 0.072) indicated no statistically significant difference in the overall distribution of coping strategies between groups. Although this difference did not reach statistical significance, the observed pattern suggests that individuals with bruxism may rely more on emotion-focused support-seeking. In contrast, those without bruxism appear to favor instrumental assistance and cognitive reframing approaches. Additionally, aggregated analysis showed that maladaptive coping strategies, including behavioral disengagement, self-blame, and substance use, were more common in the bruxism group. In contrast, adaptive strategies such as active coping, planning, and positive reinterpretation were more prevalent among controls (Figure 3).

### 4.4. GAD-7

The Mann–Whitney U test, applied due to non-normal score distributions in both groups (Shapiro–Wilk test: W = 0.956, *p* = 0.016 for the bruxism group; W = 0.923, *p* = 0.010 for the control group), showed that participants with bruxism (Group 1; *n* = 71) had significantly higher GAD-7 total scores compared with those without bruxism (Group 2; *n* = 40). The ranks were 4572 for Group 1 and 1533 for Group 2, yielding U = 713.0, Z = 4.27, and *p* < 0.00002. This indicates a markedly higher level of generalized anxiety symptoms among participants with bruxism relative to controls. Kruskal–Wallis analysis revealed a significant association between GAD-7 total scores and the duration of symptoms (H(3) = 9.50, *p* = 0.023). Post hoc tests showed that participants without symptoms (group 0) had significantly lower GAD-7 scores than those with short-term symptoms (group 1; *p* = 0.043). Mann–Whitney U tests demonstrated that participants who reported temporomandibular joint (TMJ) noises during jaw movement in the past three days had significantly higher GAD-7 scores than those without such symptoms (U = 1124.0, Z = 2.26, *p* = 0.024). Similarly, participants experiencing temporal headaches in the last 30 days scored markedly higher on the GAD-7 than those without (U = 840.5, Z = 4.00, *p* < 0.0001). These findings indicate that higher levels of generalized anxiety are linked to both the presence and the duration of pain-related symptoms, particularly TMJ noises and temporal headaches.

### 4.5. Stroop Test

Normality testing (Shapiro–Wilk) indicated non-normal distributions for all time-based Stroop indices in the control group (*p* < 0.01) and for error difference in both groups (*p* < 0.001). Mann–Whitney U tests revealed that three time-based variables, task time difference, relative index, and time ratio were significantly worse in the bruxism group compared with controls (U = 832.5, Z = 2.52, *p* = 0.012; U = 908.5, Z = 1.98, *p* = 0.047 for both indices). These results indicate longer completion times and greater interference effects in individuals with bruxism. No significant differences were observed between groups for the number of errors (U = 1045.5, *p* = 0.31), suggesting that accuracy was comparable despite slower task performance in the bruxism group. Spearman correlations showed strong interdependence among the time-based Stroop indices (ρ ≈ 0.93 between the relative index and the time ratio), but only weak associations with health status. The data suggest that bruxism is associated with reduced processing speed and inhibitory control, but not with increased error rates. Spearman’s rank correlation analysis revealed that only the Stroop task time difference was significantly correlated with GAD-7 scores (ρ = 0.166, *p* < 0.05), indicating a weak but positive association between slower interference resolution and higher generalized anxiety levels. Other Stroop indices, relative index, time ratio, and error difference, showed no significant correlations with GAD-7, suggesting that anxiety severity is more closely related to processing speed than accuracy or proportional interference measures. The analysis revealed statistically significant differences in both muscle pain levels and anxiety severity (GAD-7) depending on the field of study. The Kruskal–Wallis test for the total number of painful muscles (H(2) = 12.33, *p* = 0.0021) showed that physiotherapy students reported the highest levels of muscle pain (mean rank = 70.40), followed by dentistry students (mean rank = 62.52). In contrast, students in biological renewal (rehabilitation/wellness) reported the lowest muscle pain (mean rank = 43.18). Post hoc pairwise comparisons indicated statistically significant differences between biological renewal and physiotherapy (*p* = 0.0446) and between biological renewal and dentistry (*p* = 0.0079). In contrast, the difference between physiotherapy and dentistry was not statistically significant (*p* = 1.0000). For anxiety levels measured with the GAD-7 scale, the Kruskal–Wallis test (H(2) = 6.69, *p* = 0.0352) revealed that physiotherapy students had the highest anxiety scores (mean rank = 73.50), followed by dentistry students (mean rank = 58.72), with the lowest scores observed among biological renewal students (mean rank = 47.31). The most significant difference, approaching statistical significance, was found between physiotherapy and biological renewal (*p* = 0.0572), while all other comparisons showed no statistically significant differences (*p* > 0.22). These findings suggest that the field of study may have a meaningful impact on the frequency and intensity of muscle pain, with physiotherapy and dentistry students experiencing significantly more muscle pain than biological renewal students. In contrast, while there is some indication that the field of study may also influence anxiety levels, the observed differences, particularly between physiotherapy and biological renewal, were weaker and did not consistently reach conventional levels of statistical significance.

**Figure 4:**
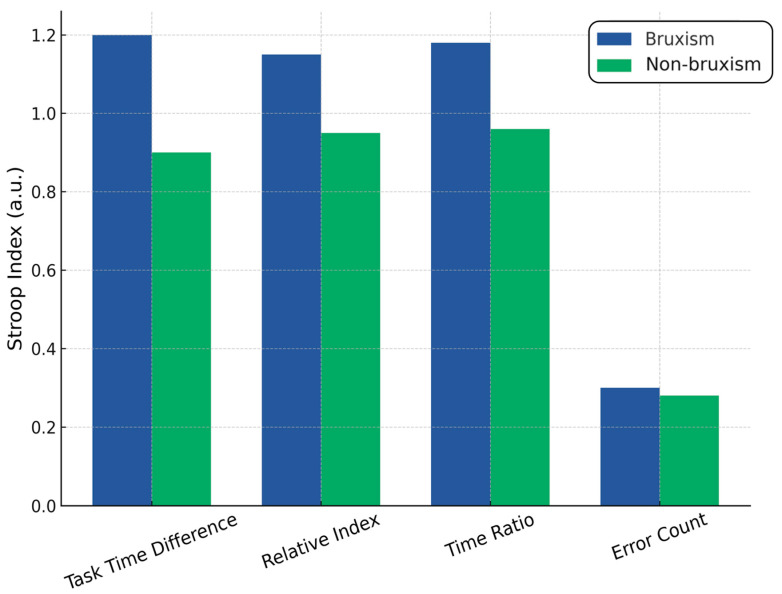
Stroop Test results in bruxism vs. non-bruxism.

The multiple regression analyses examining Stroop test performance outcomes (task time difference, relative index, time ratio, and error count difference) to anxiety levels (GAD-7) and a stress/bruxism factor revealed consistently weak associations and low explanatory power. For task time difference, the model showed a weak correlation (R = 0.235) and explained only 5.5% of the variance (R^2^ = 0.055, adjusted R^2^ = 0.036), with the overall model approaching but not reaching statistical significance (F(2,97) = 2.844, *p* = 0.063). Anxiety had no significant effect (*p* = 0.406), while stress/bruxism showed a positive trend toward significance (*p* = 0.088), suggesting a possible but unconfirmed influence. For both the relative index and time ratio measures, results were nearly identical (R = 0.146, R^2^ = 0.021, adjusted R^2^ = 0.001, F(2,97) = 1.053, *p* = 0.353), with neither predictor significantly affecting outcomes (all *p* > 0.15), indicating negligible explanatory value. For error count difference, the model again showed a weak correlation (R = 0.217, R^2^ = 0.047, adjusted R^2^ = 0.027, F(2,97) = 2.390, *p* = 0.097), with stress/bruxism demonstrating a borderline negative association (*p* = 0.054) and anxiety showing a borderline positive association (*p* = 0.097), both potentially relevant but not statistically confirmed. Taken together, these results indicate that anxiety levels do not significantly predict Stroop performance in any metric. At the same time, stress/bruxism may have a small, borderline impact on time difference and error count, warranting further investigation with larger samples (Figure 4).

### 4.6. Interpretation

Overall, the regression results indicate that neither anxiety (GAD-7) nor the stress/bruxism factor significantly predicted Stroop task performance across the examined measures (task time difference, relative index, time ratio, and error count difference), and the models generally explained only a small proportion of the variance. The weak correlations (R ranging from 0.146 to 0.235) and low R^2^ values (2–5.5%) suggest that these psychological and physiological factors have only a limited direct influence on cognitive control performance in this sample. Several factors could explain these findings. First, Stroop performance may be influenced by a broader range of cognitive, attentional, and neurological variables, such as working memory capacity, processing speed, or fatigue, which were not included in the models. Second, the potential effects of anxiety or bruxism on cognitive control might be subtle and require a larger sample size to detect, especially if these relationships are non-linear or interact with other unmeasured variables (e.g., sleep quality, coping strategies). Third, the sample characteristics, such as a relatively homogeneous population in terms of age, education, or health status, might have reduced variability in both predictors and outcomes, limiting statistical power. Finally, task-specific factors such as test length, difficulty, or participant familiarity with similar tasks could have attenuated the observable influence of psychological stress or anxiety on performance. These results therefore suggest that while anxiety and bruxism may have some clinical or experiential relevance to cognitive performance, their measurable statistical effect on Stroop task outcomes under the conditions of this study appears small.

## 5. Discussion

### 5.1. The Findings of This Study in the Context of Other Evidence

The aim of this study was to investigate the relationship between bruxism, the severity of generalized anxiety symptoms, cognitive functioning and stress coping strategies, as well as to assess the impact of the severity of masticatory muscle pain and environmental factors (field of study) on these variables [10,46]. This study provides further evidence that bruxism, particularly among students in health-related fields, is a complex condition situated at the intersection of psychological stress, coping styles, and cognitive control mechanisms. The findings support the biopsychosocial model of bruxism, in which biological, emotional, and behavioral domains interact to influence the onset and maintenance of parafunctional jaw activity [7]. The observation that participants with bruxism exhibited significantly higher GAD-7 scores than controls is consistent with prior work linking bruxism to elevated stress and anxiety levels [12]. The nearly threefold higher prevalence of clinically relevant anxiety among bruxers in our cohort mirrors patterns seen in both student and general adult populations, reinforcing the hypothesis that bruxism may be a somatic manifestation of chronic hyperarousal [47,48]. This aligns with neurobiological models implicating hypothalamic–pituitary–adrenal axis activation and increased sympathetic drive in the amplification of masticatory muscle tone [49]. The finding that students with bruxism exhibited significantly higher anxiety and a nearly threefold increase in clinically relevant anxiety supports stress physiology models of bruxism. These models emphasize dysregulation of the hypothalamic–pituitary–adrenal (HPA) axis and heightened sympathetic nervous system activity as central mechanisms. Chronic stress and anxiety can lead to sustained cortisol release and autonomic hyperactivation, which in turn increase masticatory muscle tone and contribute to repetitive jaw muscle activity. This aligns with previous studies reporting elevated salivary cortisol and increased heart rate variability preceding bruxism episodes, particularly during sleep. Our results suggest that bruxism is not merely a dental issue but a somatic manifestation of chronic hyperarousal, consistent with a biopsychosocial framework.

### 5.2. Coping Strategies and Pain Severity

An analysis of coping strategies demonstrates a shift from problem-focused to emotion-focused and avoidance-oriented coping as muscle pain severity increased [12]. Although the overall distribution of coping strategies did not differ significantly between bruxism and non-bruxism groups, the pattern suggests that bruxers rely more heavily on emotional support-seeking [12,50]. In contrast, controls tend to favor instrumental support and positive reinterpretation. These results are consistent with previous studies indicating that individuals with AB may use adaptive coping strategies [51]. There may be a bidirectional relationship between pain and coping style, in which ineffective strategies may exacerbate stress and indirectly cause pain, and persistent pain may impair the use of adaptive strategies [52]. The integration of anxiety assessment, coping evaluation, and cognitive testing provides a multidimensional profile of bruxism that extends beyond traditional dental symptomatology. Clinically, these findings highlight the need for a multidisciplinary approach to management that involves dentists, psychologists, and physiotherapists working together. Dentists can address, e.g., occlusal appliance therapy and work with muscles hyperactivity, psychologists and psychotherapists can implement stress-reduction interventions such as coping-skills training or psychotherapy (cognitive-behavioral CBT, Gestalt therapy or others). Physiotherapists can help alleviate muscle tension, improve jaw function, and also teach how to exercise at home. Such coordinated care pathways may prevent the progression of bruxism to chronic pain, sleep disorders, and long-term functional impairment. Incorporating simple psychological screening tools like the GAD-7 and COPE Inventory into routine dental or medical visits could help identify at-risk individuals early and facilitate timely referral to appropriate specialists. Furthermore, by addressing the psychosocial dimensions of bruxism, such interventions may also reduce the burden of anxiety and stress commonly associated with the disorder while simultaneously enhancing patients’ self-awareness regarding their own condition.

### 5.3. Executive Function Findings and the Role of the DLPFC

One novel aspect of this study is the inclusion of Stroop task performance as a proxy for dorsolateral prefrontal cortex (DLPFC) inhibitory control. Bruxism participants demonstrated significantly greater Stroop interference times, indicating slower cognitive inhibition, but without a corresponding increase in errors [53]. This suggests that bruxers may preserve accuracy at the expense of speed when faced with competing cognitive demands. The weak correlations between GAD-7 scores and interference times, along with low explanatory power in regression models, indicate that anxiety alone does not fully account for inhibitory control differences [54]. This suggests that the observed differences in cognitive functions are multifactorial and largely independent of the level of anxiety or the presence of bruxism. Variables not included in the model, such as sleep quality, fatigue level, individual differences in cognitive resources, or lifestyle, may also play a role [55]. Instead, these results may reflect a broader interplay between stress physiology, attentional control, and motor regulation, as proposed in neurocognitive models of bruxism [56].

### 5.4. Field of Study as a Modulator of Risk

An interesting secondary finding was that physiotherapy and dentistry students reported masticatory muscle pain more frequently compared to students in the biological renewal program, indicating that a higher proportion of students in these two fields experienced muscle pain than their peers in biological renewal.

This may reflect differences in academic workload, clinical exposure, or personality traits associated with specific professional tracks. The implication is that professional training environments could contribute to bruxism risk through sustained psychosocial stressors [57,58,59].

### 5.5. Clinical and Research Implications

The integration of anxiety assessment (GAD-7), coping evaluation (COPE), and cognitive control testing (Stroop) offers a multidimensional profile of bruxism that extends beyond dental symptomatology and highlights the need for interdisciplinary care. Clinically, this suggests that management strategies should combine stress reduction interventions such as mindfulness, relaxation training, and psychotherapy with pain management protocols that address both muscular symptoms and their psychological consequences, as well as cognitive training or neurofeedback approaches aimed at enhancing inhibitory control in patients with evidence of slowed cognitive interference resolution. This comprehensive approach may improve treatment effectiveness, accelerate recovery, reduce costs, and prevent long-term complications such as chronic facial pain, temporomandibular joint disorders, excessive muscle tension, sleep disturbances, and cognitive decline. Future studies should incorporate objective verification of bruxism using polysomnography or electromyography, employ diverse tools for assessing emotional regulation and stress, and explore causal mechanisms linking stress, coping strategies, and bruxism symptoms in varied populations beyond academic settings. Our findings also suggest that psychological screening tools like the GAD-7 and COPE Inventory could be incorporated into routine dental and medical assessments to identify individuals at risk for bruxism-related complications at an early stage. These simple, self-administered measures require minimal time and resources, yet they provide valuable insights into psychoemotional factors contributing to bruxism; for example, elevated GAD-7 scores or predominant emotion-focused and avoidance coping strategies could alert clinicians to psychosocial stressors underlying muscle pain or temporomandibular symptoms. Integrating such tools into dental examinations, student health screenings, or primary care visits would enable early interdisciplinary referral for stress management or behavioral interventions, aligning with the biopsychosocial model and supporting comprehensive care that extends beyond symptomatic dental treatment.

### 5.6. Limitations of Our Study

This study has several limitations that should be considered when interpreting the findings. Its cross-sectional design precludes conclusions about causal relationships between bruxism, anxiety, coping strategies, and inhibitory control, and longitudinal studies are needed to establish temporal relationships and potential causal pathways. The sample consisted primarily of young health sciences students, which limits generalizability to broader and more diverse populations, such as older adults, non-academic groups, or individuals with different cultural or socioeconomic backgrounds. Bruxism was diagnosed using a combination of standardized clinical examination and self-report, consistent with the 2018 international consensus for probable bruxism; however, polysomnography (PSG) and electromyography (EMG), the gold standard methods for definitive diagnosis, were not used, which may have led to some misclassification despite the use of two convergent approaches. Although the Stroop task provided valuable insights into inhibitory control, it captured only one domain of executive function, leaving other cognitive processes such as working memory, sustained attention, or cognitive flexibility unexplored. Several potential confounders, including sleep quality, fatigue, caffeine or nicotine use, medication intake, circadian rhythm, and prior traumatic experiences, were not systematically controlled, all of which could influence both bruxism expression and cognitive performance. Additionally, while the psychological instruments used were validated, they may not have fully captured nuanced aspects of emotional regulation or stress reactivity. Collectively, these limitations indicate that while the present findings highlight meaningful associations, further studies with larger and more diverse samples, objective multimodal bruxism diagnostics, and broader cognitive and psychosocial assessments are required to strengthen causal inference and improve external validity. Finally, because our sample size justification was conducted post hoc rather than before recruitment, there is a risk that the study may have been under- or overpowered for certain secondary analyses (Table 4).

## 6. Conclusions

Bruxism among health sciences students is closely linked to elevated anxiety, maladaptive coping strategies, and subtle impairments in cognitive inhibition. Students with bruxism showed higher GAD-7 scores, more frequent headaches and temporomandibular symptoms, and a shift from problem-focused toward emotion-focused and avoidance coping. Stroop results suggest slower inhibitory responses despite preserved accuracy. Physiotherapy and dentistry students reported more anxiety and muscle pain than students in biological renewal, reflecting differences in academic stress. These findings support viewing bruxism as a multifactorial biopsychosocial condition and highlight the need for interdisciplinary management that combines dental care with psychological assessment and stress reduction strategies. Future studies using objective diagnostic tools and larger, more diverse populations are warranted to clarify causal mechanisms and guide tailored interventions. Future longitudinal, multi-center studies using objective diagnostic tools such as polysomnography or electromyography are essential to confirm these findings and clarify causal pathways.

## Figures and Tables

**Table 1:** The selection criteria for patient qualification in the study.

Inclusion Criteria	Exclusion Criteria
1. Written informed consent to participate	1. Diagnosed neurological disorders unrelated to bruxism
2. Age between 18 and 60 years	2. Severe psychiatric conditions
3. Medically stable with no uncontrolled acute or chronic conditions	3. Current medications affecting central nervous system functions
4. Recent significant dental or orthodontic treatment (within six months prior)	
5. Use of analgesic drugs and/or drugs affecting muscle function	
6. Disease or autoimmune disorder associated with generalized muscular tension	

**Table 2:** Demographic Characteristics of Study Participants.

Median (Range)	n (%) or M ± SD	Characteristic
		Sex
22.5 (18–37)	74 (66.6%)	Women
21.5 (18–31)	37 (33.3%)	Men
22 (18–37)	22.29 ± 3.10	Age (years)

**Table 3:** Distribution of Participants diagnosed with Bruxism by Gender, Age, and Field of Study.

Percentage	Subcategory	Category
66.6%	Women	**Gender**
33.3%	Men	
91.8%	18–26	**Age Group**
6.4%	27–30	
1.8%	31–40	
17.8%	19	**Specific Age**
16.9%	23	
15.7%	24	
13.5%	22	
12.2%	20	
5.1%	5.1%	21
4.1%	25	
3.2%	26	
5.5%	27	
2.4%	2.4%	30
50.9%	Dentistry	**Field of Study**
40.0%	Biological Renewal	
9.1%	Physiotherapy	

**Table 4:** Summary of key indications, findings, and clinical implications.

Clinical Implication	Key Finding	Indication
High burden in students. Routine screening is justified in university clinics and student health.	Bruxism 63.96% (71/111)	Prevalence
Add brief GAD-7 screening to dental and primary care intakes. Early referral to psychology when elevated.	Higher in bruxism vs. controls: 10.63 ± 5.78 vs. 5.80 ± 3.66; mean diff 4.83; g 0.94; *p* < 0.001. Clinically relevant anxiety 55.4% vs. 19.6% (RR 2.83)	Anxiety (GAD-7)
Ask targeted questions about recent headache and TMJ noises to flag anxiety-related risk and bruxism activity.	Temporal headache linked to higher GAD-7; TMJ noises associated with higher GAD-7	Headache and TMJ noises
Consider counseling on stress management and, when available, cognitive training or neurofeedback for inhibitory control.	Slower interference in bruxism: 42.19 ± 16.87 s vs. 34.57 ± 16.25 s; mean diff 7.63 s; g 0.46; *p* = 0.027; accuracy similar	Cognitive inhibition (Stroop)
Use COPE to identify maladaptive strategies. Offer brief coping-skills training or refer for CBT.	Shift toward emotion-focused and avoidance with increasing muscle pain; controls favored active coping and planning	Coping style (COPE)
Combine dental pain management with psychoeducation and coping-skills interventions.	Greater maladaptive coping with higher masticatory muscle pain; severe pain linked to behavioral disengagement	Pain severity
Prioritize screening and prevention in high-stress programs. Coordinate with program health services.	Physiotherapy and dentistry students reported more muscle pain than biological renewal students	Field of study
Acknowledge possible misclassification. Use multimodal confirmation in research and complex cases.	Probable bruxism via DC/TMD exam + self-report. No PSG or EMG	Diagnostic approach

## Data Availability

The original contributions presented in the study are included in the article; further inquiries can be directed to the corresponding author.

## References

[1] Minervini G., Franco R., Di Blasio M., Martelli M., Gargari M., Bollero P., Cicciù M. (2025). Prevalence of bruxism in patients affected by epilepsy: A systematic review and meta-analysis. Acta Odontol. Scand..

[2] Maeda-Iino A., Osako Y., Nakagawa S., Takahashi K., Oga Y., Furukawa-Sainoki M., Harada M., Fukushima M., Miyawaki S. (2023). Relationship between masseter muscle activity during wakefulness and temporomandibular disorder-related symptoms. J. Oral Rehabil..

[3] Lobbezoo F., Ahlberg J., Raphael K.G., Wetselaar P., Glaros A.G., Kato T., Santiago V., Winocur E., De Laat A., De Leeuw R. (2018). International consensus on the assessment of bruxism: Report of a work in progress. J. Oral Rehabil..

[4] Verhoeff M.C., Lobbezoo F., Ahlberg J., Bender S., Bracci A., Colonna A., Fabbro C.D., Durham J., Glaros A.G., Häggman-Henrikson B. (2025). Updating the Bruxism Definitions: Report of an International Consensus Meeting. J. Oral Rehabil..

[5] Wieckiewicz M., Paradowska-Stolarz A., Wieckiewicz W. (2014). Psychosocial aspects of bruxism: The most paramount factor influencing teeth grinding. BioMed Res. Int..

[6] Koecklin K.H.U., Castillo A.A.-D., Li P. (2024). The neural substrates of bruxism: Current knowledge and clinical implications. Front. Neurol..

[7] Flueraşu M.I., Bocşan I.C., Țig I.A., Iacob S.M., Popa D., Buduru S. (2022). The Epidemiology of bruxism in relation to Psychological Factors. Int. J. Environ. Res. Public Health.

[8] Lee Y.H., Chon S., Auh Q.S., Verhoeff M.C., Lobbezoo F. (2025). Clinical, psychological, and hematological factors predicting sleep bruxism in patients with temporomandibu-lar disorders. Sci. Rep..

[9] AlSahman L., AlBagieh H., AlSahman R. (2024). Is There a Relationship between Salivary Cortisol and Temporomandibular Disorder: A Systematic Review. Diagnostics.

[10] Soto-Goñi X.A., Alen F., Buiza-González L., Marcolino-Cruz D., Sánchez-Sánchez T., Ardizone-García I., Aneiros-López F., Jiménez-Ortega L. (2020). Adaptive stress coping in awake bruxism. Front. Neurol..

[11] Minakuchi H., Fujisawa M., Abe Y., Iida T., Oki K., Okura K., Tanabe N., Nishiyama A. (2022). Managements of sleep bruxism in adult: A systematic review. Jpn. Dent. Sci. Rev..

[12] Saczuk K., Lapinska B., Wilmont P., Pawlak L., Lukomska-Szymanska M. (2019). Relationship between Sleep Bruxism, Perceived Stress, and Coping Strategies. Int. J. Environ. Res. Public Heal..

[13] Naber M., Vedder A., Brown S.B.R.E., Nieuwenhuis S. (2016). Speed and lateral inhibition of stimulus processing contribute to individual differences in Stroop-Task performance. Front. Psychol..

[14] Mesko M.E., Hutton B., Skupien J.A., Sarkis-Onofre R., Moher D., Pereira-Cenci T. (2017). Therapies for bruxism: A systematic review and network meta-analysis (protocol). Syst. Rev..

[15] Shetty S., Pitti V., Babu C.L.S., Kumar G.P.S., Deepthi B.C. (2010). Bruxism: A Literature Review. J. Indian Prosthodont. Soc..

[16] Huang M., Liang X., Lin X., Li W., Jiang Y., Geng F. (2025). Amygdala-Dependent Signaling Pathways and Regulating Circuits in Fear Memory. Synapse.

[17] Gizler M., Pietrzak N., Saczuk K., Lukomska-Szymanska M., Lapinska B. (2023). Students’ awareness of the bruxism causes, effects and therapies. Heliyon.

[18] Archer A.B., Da-Cas C.D., Valesan L.F., Cunha T.C.A., Januzzi E., Garanhani R.R., Canales G.d.L.T., de Souza B.D.M. (2023). Prevalence of awake bruxism in the adult population: A systematic review and meta-analysis. Clin. Oral Investig..

[19] Ahlberg J., Savolainen A., Rantala M., Lindholm H., Könönen M. (2004). Reported bruxism and biopsychosocial symptoms: A longitudinal study. Community Dent. Oral Epidemiol..

[20] Mekari S., Murphy R.J.L., MacKinnon A.R.S., Hollohan Q., Macdougall S.C., Courish M.K., Kimmerly D.S., Neyedli H.F. (2022). The impact of a short-period head-down tilt on executive function in younger adults. Sci. Rep..

[21] Xu X., Deng Z.-Y., Huang Q., Zhang W.-X., Qi C.-Z., Huang J.-A. (2016). Prefrontal cortex-mediated executive function as assessed by Stroop task performance associates with weight loss among overweight and obese adolescents and young adults. Behav. Brain Res..

[22] Manfredini D., Colonna A., Bracci A., Lobbezoo F. (2019). Bruxism: A summary of current knowledge on aetiology, assessment and management. Oral Surg..

[23] Taravati S., Ataeian F., Mofradnejad S., Rakhshan V. (2025). Risk factors for dental anxiety and cooperativeness in pediatric patients with or without oral habits (bruxism, nail biting, and thumb sucking). BMC Oral Health.

[24] Famuyiro K., Bruxism (Teeth Grinding): Causes, Diagnosis and Treatments—Los Angeles Times Los Angeles Times, 13 August 2025. https://www.latimes.com/dentistry/general/preventive/story/bruxism-teeth-grinding-causes-diagnosis-treatments.

[25] Saricam E., Tayman M.A. (2025). Bruxism assessment combining fractal analysis, clinical evaluation, and self-reports: A case-control study. BMC Oral Health.

[26] Schneider U.E.M., Moser L. (2024). Achieving excellence with interdisciplinary approaches in complex orthodontic adult patients. Br. Dent. J..

[27] Bastos T., Pinto R., Dias I., Leite I., Leite F. (2018). Self-care treatment on patients with wakefulness bruxism. Int. J. Orofac. Myol..

[28] Baron S. (2023). Bruksizm i Jego Powikłania.

[29] Saracutu O.I., Pollis M., Bulferetti L.B., Kaya E., Cagidiaco E.F., Ferrari M., Colonna A., Manfredini D. (2025). Comparison between two different registration protocols for the count of sleep bruxism events in a sample of healthy individuals. Clin. Oral Investig..

[30] Colonna A., Thomas D.C., Do T.T., Manfredini D. (2024). Sleep Disorders Affecting Prognosis of Dental Treatment. Dent. Clin. North Am..

[31] Manfredini D., Winocur E., Guarda-Nardini L., Paesani D., Lobbezoo F. (2013). Epidemiology of bruxism in adults: A systematic review of the literature. J. Orofac Pain.

[32] Pollis M., Lobbezoo F., Saracutu O.I., Colonna A., Manfredini D. (2025). Psychological Distress: A Mediating Factor in the Relationship Between Sleep Bruxism and Tobacco Smoking. J. Oral Rehabil..

[33] Kato T., Rompré P., Montplaisir J.Y., Sessle B.J., Lavigne G.J. (2001). Sleep bruxism: An oromotor activity secondary to micro-arousal. J. Dent. Res..

[34] Lavigne G., Kato T., Kolta A., Sessle B.J. (2003). Neurobiological mechanisms involved in sleep bruxism. Crit. Rev. Oral Biol. Med..

[35] Wieczorek T., Jodkowska A., Orzeszek S., Wieckiewicz M., Michalek-Zrabkowska M., Mazur G., Rymaszewska J., Smardz J., Wojakowska A., Martynowicz H. (2024). Why am I grinding and clenching? Exploration of personality traits, coping strategies, oral parafunctional behaviors, and se-vere sleep bruxism in a polysomnographic study. Front. Psychiatry.

[36] Trindade M., Orestes-Cardoso S., de Siqueira T.C. (2015). Interdisciplinary treatment of bruxism with an occlusal splint and cognitive behavioral therapy. Gen. Dent..

[37] Osiewicz M., Ciapała B., Bolt K., Kołodziej P., Więckiewicz M., Ohrbach R. (2024). Diagnostic Criteria for Temporomandibular Disorders (DC/TMD): Polish assessment instruments. Dent. Med. Probl..

[38] Carver C.S., Scheier M.F., Weintraub J.K. (1989). Assessing coping strategies: A theoretically based approach. J. Personal. Soc. Psychol..

[39] Juczyński Z., Ogińska-Bulik N. (2009). Narzędzia Pomiaru Stresu i Radzenia Sobie ze Stresem.

[40] Spitzer R.L., Kroenke K., Williams J.B.W., Löwe B. (2006). A brief measure for assessing generalized anxiety disorder. Arch. Intern. Med..

[41] Basińska B.A., Kwissa-Gajewska Z. (2023). Psychometric properties of the Polish version of the Generalized Anxiety Disorder Scale (GAD-7) in a non-clinical sample of employees during pandemic crisis. Int. J. Occup. Med. Environ. Health.

[42] Stroop J.R. (1935). Studies of interference in serial verbal reactions. J. Exp. Psychol..

[43] Golden C.J. (1978). Stroop Color and Word Test: A Manual for Clinical and Experimental Uses.

[44] Cohen J.D., Dunbar K., McClelland J.L. (1990). On the control of automatic processes: A parallel distributed processing account of the Stroop effect. Psychol. Rev..

[45] Posner M.I., Snyder C.R., Solso R.L. (1975). Attention and cognitive control. Information Processing and Cognition: The Loyola Symposium.

[46] Chemelo V.D.S., Né Y.G.S., Frazão D.R., de Souza-Rodrigues R.D., Fagundes N.C.F., Magno M.B., da Silva C.M.T., Maia L.C., Lima R.R. (2020). Is There Association Between Stress and Bruxism? A Systematic Review and Meta-Analysis. Front Neurol..

[47] Ahlberg J., Lobbezoo F., Ahlberg K., Manfredini D., Hublin C., Sinisalo J., Kononen M., Savolainen A. (2013). Self-reported bruxism mirrors anxiety and stress in adults. Med. Oral Patol. Oral Cir. Bucal..

[48] Papadimitriou A., Priftis K.N. (2009). Regulation of the Hypothalamic-Pituitary-Adrenal axis. NeuroImmunoModulation.

[49] García F.E., Barraza-Peña C.G., Wlodarczyk A., Alvear-Carrasco M., Reyes-Reyes A. (2018). Psychometric properties of the Brief-COPE for the evaluation of coping strategies in the Chilean population. Psicol. Reflex. Crit..

[50] Bracci A., Lobbezoo F., Colonna A., Bender S., Conti P.C.R., Emodi-Perlman A., Häggman-Henrikson B., Klasser G.D., Michelotti A., Lavigne G.J. (2023). Research routes on awake bruxism metrics: Implications of the updated bruxism definition and evaluation strategies. J. Oral Rehabil..

[51] Weeratunga E., Senadheera C., Hettiarachchi M., Perera B. (2022). Validation of the Sinhalese Version of Brief COPE Scale for patients with cancer in Sri Lanka. BMC Psychol..

[52] Perrotta D., Bianco V., Berchicci M., Quinzi F., Perri R.L. (2021). Anodal tDCS over the dorsolateral prefrontal cortex reduces Stroop errors. A comparison of different tasks and designs. Behav. Brain Res..

[53] Laskov O., Biačková N., Stuchlíková Z., Kostýlková L., Klírová M. (2023). Inhibitory Control in Young Healthy Adults—A tDCS Study. Physiol. Res..

[54] Wu D., Zhou Y., Xu P., Liu N., Sun K., Xiao W. (2022). Initial performance modulates the effects of cathodal transcranial direct current stimulation (tDCS) over the right dorsolateral prefrontal cortex on inhibitory control. Brain Res..

[55] Pavlou I.A., Spandidos D.A., Zoumpourlis V., Papakosta V.K. (2024). Neurobiology of bruxism: The impact of stress (Review). Biomed. Rep..

[56] Kunzler A.M., Helmreich I., König J., Chmitorz A., Wessa M., Binder H., Lieb K. (2020). Psychological interventions to foster resilience in healthcare students. Cochrane Database Syst. Rev..

[57] Gallasch D., Conlon-Leard A., Hardy M., Phillips A., Van Kessel G., Stiller K. (2022). Variable levels of stress and anxiety reported by physiotherapy students during clinical placements: A cohort study. Physiotherapy.

[58] Driscoll M.A., Edwards R.R., Becker W.C., Kaptchuk T.J., Kerns R.D. (2021). Psychological interventions for the treatment of chronic pain in adults. Psychol. Sci. Public Interest.

[59] Bin Rahmah A.S., Alsaif M.I., Naser A.Y. (2025). The Complex Interplay Between Dental Anxiety, Generalized Anxiety, and Dental Neglect and Oral Health Quality of Life in the General Public. Healthcare.

